# Impairment Assessment in Adult ADHD and Related Disorders: Current Opinions From Clinic and Research

**DOI:** 10.1177/10870547241261598

**Published:** 2024-06-19

**Authors:** Anselm B. M. Fuermaier, Caroline Gontijo-Santos Lima, Oliver Tucha

**Affiliations:** 1 University of Groningen, The Netherlands; 2 University Medical Center Rostock, Germany; 3 National University of Ireland, Maynooth, Ireland

**Keywords:** adult ADHD, assessment, diagnosis, functional impairment, functional outcome

## Abstract

**Objectives:**

Assessing functional impairment is one of the essential components in the clinical evaluation of ADHD in adulthood, serving both diagnostic and outcome evaluation purposes. However, clinicians and researchers may face challenges in selecting suitable instruments due to variations in accessibility and quality of instruments.

**Methods:**

We conducted an online survey involving an international group of 92 respondents engaged in clinical practice and/or research on ADHD. The survey aimed to evaluate current practices in assessing impairment in adult ADHD and related disorders, while also identifying areas requiring adaptation or potential new developments.

**Results:**

Our findings revealed that clinicians and researchers utilize a diverse range of instruments for assessing impairment in adults with ADHD, including some that may lack adequate properties for this purpose. Notably, dissatisfaction with current practice standards was expressed, underscoring the need for novel assessment approaches and improved psychometric properties.

**Conclusion:**

It is evident that research endeavors are warranted to either refine existing measures or devise new ones for assessing functional impairment in adult ADHD. Emphasis should be placed on disseminating instruments that enhance accessibility in both research and clinical settings, and facilitate streamlined administration and interpretation.

## Introduction

ADHD is a neurodevelopmental childhood disorder with a fluctuating course of remission and recurrence over time. About 90% of children with ADHD continue to struggle with residual ADHD in young adulthood (Sibley et al., 2022), whereas the worldwide prevalence of persistent adult ADHD was estimated to be 2.58% in a recent meta-analysis (Song et al., 2021). International diagnostic classification systems (i.e., DSM-5, American Psychiatric Association [APA], 2013; and ICD-11, World Health Organization, 2022) define ADHD by symptoms of inattention, hyperactivity, and impulsivity. In addition to the symptom criteria, classification systems of ADHD require that symptoms result in significant impairments in more than one setting of functioning. For ADHD in adulthood, typical impairments include problems in finishing education, poor grades in university education programs (e.g., Advokat et al., 2011; Arnold et al., 2020), job fluctuations, and dissatisfaction (Fuermaier et al., 2021), problems in interpersonal relationships and romantic relationship breakups (e.g., Bruner et al., 2015; Michielsen et al., 2015), maladaptive parenting, financial difficulties (Bangma et al., 2019; Koerts et al., 2023), misuse of alcohol and drugs (Van Emmerik-van Oortmerssen et al., 2012), and unsafe car driving and rule violations (e.g., Fuermaier et al., 2017; see Sibley, 2021, for diagnostic issues in adult ADHD).

The assessment of impairment in every given ADHD evaluation can be seen as a gatekeeper that protects against over-diagnosis. In contrast, the presence of ADHD symptoms without the experience of functional impairment does not fulfill the criteria of a mental disorder (Spitzer et al., 2018) and represents a serious threat for diagnostic validity and common reason for false positives (Sibley, 2021). A recent meta-analysis of Harrison and Edwards (2023) evidently demonstrated that screening for ADHD symptoms is not sufficient to establish a diagnosis of adult ADHD but results in an unacceptably large number of false positives. Whilst ADHD screeners were useful to identify true ADHD cases (negative predictive values > 96%), positive predictive values remained below 20% for most studies reviewed. The crucial contribution of impairment assessment to the diagnostic accuracy of adult ADHD is underlined by studies who showed that 77% (Gordon et al., 2006) or 41% (Gathje et al., 2008) of individuals who were screened for ADHD on a symptom checklist failed to meet the impairment criterion of ADHD. To this end, the high rates of ADHD diagnoses and public debate about overdiagnoses may, at least in parts, reflect incomplete diagnostic evaluations (Johnson & Suhr, 2021). And indeed, research shows that about three quarters of clinicians miss any diagnostic criteria in their assessments, and still more than the half of all clinicians overlook the impairment criterion (Nelson et al., 2014; Weis et al., 2019). Hence, the literature reviewed above suggests that currently applied diagnostic procedures are at risk of bypassing diagnostic criteria that are required to make a well-informed diagnosis of ADHD, including and foremost the criteria of significant impairment.

Furthermore, impairment assessment in adult ADHD is not only important for diagnostic reasons, but also for treatment outcome evaluation. This is relevant because symptomatic response and functional response are not always consistent with one another, but a substantial proportion of patients remain functionally impaired even after part or full symptom remission (Sasser et al., 2016; Weiss, 2022). This stands in contrast to research practice, in which the vast majority of studies focus on symptoms and diagnosis, while only a substantial smaller body of research addresses functional outcome (Hoagwood et al., 2012). A systematic measurement of both symptoms and functional impairment is claimed prominently (Weiss, 2022), because full remission requires the normalization of both symptoms and function (Biederman et al., 2000) and we otherwise fail to identify and treat the true difficulties of our clients. For this purpose, clinicians and researchers require efficient and effective tools that indicate impairment compared to the general population at baseline, but that are also sensitive to indicate change in impairment from baseline to post-treatment. Objective indication of real-world evidence of dysfunctioning is one option to measure impairment (e.g., university grades, job fluctuations, marriage breakups, financial issues such as depts or gambling losses, and traffic violation penalties), but this type of information is often cumbersome to access, sensitive to context and therefore hard to compare across time and individuals. Several psychometrically sound and empirically proven instruments are available for clinic and research in children, adolescents, and adults with ADHD (Sasser et al., 2016; Weiss, 2022).

In the impairment assessment, one need to differentiate between absolute impairment (lifelong and relatively stable, static trait) and relative impairment (functional impairment secondary to a diagnosis or symptoms). The differentiation is relevant for clinic and research, because relative functional impairment may improve without large measurable delay, while absolute functional impairment may only change very slowly over time (for a discussion, see Weiss, 2022). The tools available for adults are mostly self-report in nature, which should be accompanied by information from significant others to corroborate patients’ self-reports. A meaningful contribution toward a conceptual basis for a comprehensive impairment assessment in adult ADHD is provided by the WHO’s International Classification of Functioning, Disability and Health (ICF) core sets (Bölte et al., 2018, 2024; De Schipper, Mahdi, et al., 2015; de Schipper, Lundequist, et al., 2015; Mahdi et al., 2018). Systematic and thorough empirical work on ICF core sets made significant milestones toward raising awareness and recognition, reaching consensus on a conceptual framework, and defining relevant domains of functioning.

However, despite the compelling arguments and evidence for adequate assessment of functional impairment in adult ADHD, it appears to be missing in many contexts of clinic and research, which may indicate the presence of obstacles for clinicians and researchers to refrain from, entirely or in parts, a comprehensive functional impairment assessment. Potential reasons could be a misconception of what functional impairment entails, a lack of knowledge about or access to adequate instruments, uncertainty on their administration and interpretation, or dissatisfaction regarding their properties for use in research and clinical practice.

On the grounds of the importance of impairment assessment, but the inconsistency in the attention it receives in practice, the goal of the present study was to consult an international group from research and clinical practice. In this survey, we aimed to learn about their understanding of the concept of functional impairment in adult ADHD, the purpose of its assessment, current clinical standard in the impairment assessment, and about a potential need for adjustment and new developments. This study should help to explain the current discrepancy between the need for functional impairment assessment and its application in practice, identify most useful tools and approaches, and inform future research developments for a better impairment assessment in adults with ADHD and related disorders.

## Methods

### Participants

We considered potential participants for inclusion in our survey (see Table 1 for the complete survey) if they had clinical and/or research experience in the field of ADHD and related disorders. Potential participants were approached in personal communication, by email, or via third persons in their working environment. Additionally, potential participants in adult ADHD assessment were systematically approached at the Ninth World Congress ADHD, May 18 to 21, 2023, in Amsterdam, the Netherlands. The World Congress ADHD is the worldwide largest expert meeting on ADHD and attracts clinicians and researchers from all over the globe to exchange on this topic. Participation in this survey was voluntary and unpaid. Initially, we considered data of 142 participants for inclusion in our study. After removal of responses of participants who did not consent to process personal data or who did not provide any data after descriptive information (*n* = 48), and those who did not have any clinical experience with ADHD and research output in the field (*n* = 2), we retained responses from 92 participants, for data analysis. given between the 7th of March and 18th of September, 2023. Table 2 presents participants’ characteristics regarding age, gender, place of residence, discipline and degree of education, and clinical and research output. It must be noted that not all participants responded to all sections and questions of this survey, which resulted in varying number of responses (see Table 1 for number of valid responses per question, and corresponding drop outs per section).

**Table 1.** Complete Survey and Number of Responses per Question.

Questions		Valid *N*
Section 1: Descriptives		
1. Age (in years)	(open question)	92
2. Gender	Male/Female/Other	91
3. Country and place of residence	Country, place	92
4. Discipline of education	Psychology, Psychiatry, Other	92
5. Highest academic degree	BSc, MSc, MD, PhD, Prof., Other	92
6. Clinical experience with adult ADHD	0–40 years	92
7. How do you distribute your work time?	(in %) clinic, research, other	92
8. Did you publish about adult ADHD?	(# publications) 0–100+	55
Section 2: Current clinical standard		
In the following questions, please think about the clinical assessment of adults with ADHD specifically. Please also think about typical coexistent disorders next to ADHD, as adult ADHD in adulthood is highly comorbid and typically coexists with various other psychotic disorders		
1. What do you understand under the term “impairment” in this context? (Open question)		59
2. For which purpose do you assess impairment in the assessment of adult ADHD? (Multiple answers possible) • Supporting diagnosis • Determining treatment needs • Creating treatment plan • Evaluating treatment response or resistance • Establishing congruence or divergence with symptoms • other, namely:		71
3. Which instruments or approaches are you applying for the assessment of impairment in adults with ADHD? • Instrument 1:_____ • Instrument 2:_____ • Instrument 3:_____		70
4. Please rate quality of instrument 1 • Rating poor (0), mediocre (1), decent (2), good (3), excellent (4)		57
5. Please list strengths and weaknesses of instrument 1 (open question) *Questions 3–5 repeated for all instruments listed		46
Section 3: Needs for new developments		
1. Do you think there is a need for adaptations or even new developments for a better impairment assessment in adult ADHD? (multiple choice) • No, no need to change current practice (0) • A bit, minor adaptations needed (1) • Much, considerable adaptations and/or new development needed (2) • Very much, substantial adaptations and new development needed (3)		54
2. What are the most important domains of functioning in the impairment assessment in adult ADHD? You can list up to 10 domains. Per box, write down: • The domain • Is there anything that you find particularly important when assessing the respective domain? Note: The order of the boxes indicates the order of importance. You can move the order with the commonly known “drag and drop” function (open question)		45
3. Please rate the importance of the following aspects of validation, that you require from a good instrument for the assessment of impairment in adults with ADHD (Rating of irrelevant (0), little (1), much (2), and very much (3)) • Quick administration • Automatic scoring • Good population norms • Specific disorder (adult ADHD) • Sensitive to change • Association or dissociation between impairment domains • Association or dissociation to symptom severity • Association or dissociation to burden of disease • Please note further aspects of importance if missed them in list above		51
4. How suitable do you consider the following assessment approaches for the impairment assessment in adult ADHD? (Rating of poor (0), little (1), much (2), very much (3)) • Self-report • Other report • Discrepancy between sources of information, for example, self-report vs. other-report • Different scaling: Counting frequency instead of subjective severity rating • Objective indications (e.g., facts or events) instead of subjective evaluation • In-the-moment assessment (e.g., responding via apps during different times of the day) • Consideration and integration of multiple sources and factors • Please note further assessments if missed in list above:________		51

**Table 2.** Characteristics of Participants.

	*M* (*SD*)	Range	*N*
Age (in years)	44.5 (13.1)	22–88	92
	*N* (%)		*n*
Gender			
Male	34.1		31
Female	65.9		60
Place of residence			
Europe	72.2		65
North America	16.7		15
Asia	11.1		10
Highest academic degree/position			
BSc	3.3		3
MD	14.1		13
MSc	28.3		26
PhD	43.5		40
Professor	5.4		5
Other	5.4		5
Discipline of education/training			
Psychiatry	19.6		18
Psychology	72.8		67
Other	7.6		7
Clinical experience with adult ADHD (in years)			
0–5	34.4		31
6–10	23.4		21
11–20	27.8		25
21–30	10		9
31–40	4.4		3
# Publications in adult ADHD			
0–5	61.8		34
6–10	12.7		7
11–20	12.8		7
21–40	3.6		2
41+	9.1		5

*Note*. Europe = Sweden (*n* = 28), Germany (*n* = 18), Netherlands (*n* = 10), Switzerland (*n* = 2), Hungary (*n* = 2), Croatia (*n* = 1), Norway (*n* = 1), Poland (*n* = 1), Andorra (*n* = 1), Czech Republic (*n* = 1), North America = Canada (*n* = 12), USA (*n* = 3), Asia = China (*n* = 4), Israel (*n* = 4), Japan (*n* = 1), and Pakistan (*n* = 1).

### Materials and Procedure

An online questionnaire was designed on the Qualtrics survey software tool. The questionnaire was designed in English language and consisted of three parts, that is (I) descriptive information of the respondent, (II) current clinical standard in the impairment assessment, and (III) potential needs for adjustment or new developments in the impairment assessment. The design of the questions were a mixture of multiple choice and open-ended answers. A complete presentation of the survey, including its questions, answer options, order of presentation, and number of responses, is given in Table 1.

The online survey started with informing potential participants about the purpose and content of the survey, dealing with personal information, and requesting active informed consent by clicking on respective buttons. The survey flow was initiated upon agreement of participation and took about 11 minutes to complete (median = 10.45 minutes). At the end of the survey, participants were asked to indicate their email address in case they liked to be considered for potential follow-up studies on the basis of the survey results. The study protocol was reviewed and approved by [anonymized for peer review].

### Statistical Analysis

Survey questions employing a multiple-choice format were analyzed and presented in descriptive statistics. Responses to open-ended questions underwent a comprehensive manual analysis by the research team. These analyses encompassed a blend of qualitative techniques, specifically thematic analysis and qualitative content analysis. Firstly, the thematic analysis was carried out in that the entirety of the dataset of open-ended questions was reviewed to identify any discernible patterns or recurring themes. This initial phase allowed for the identification of prominent patterns, particularly within the questions on assessed impairments as well as those pertaining to strengths and weaknesses. Following this, distinct categories (see tables) were established to capture the observed themes and patterns, for use in both thematic and qualitative content analysis. Categories were refined and adjusted, and individual responses were systematically categorized based on the established themes and patterns. In the case of impairment categories, pivot tables were employed to facilitate further analysis, while a conventional summary table was utilized for summarizing strengths and weaknesses. Subsequently, the categorized data and analysis were subjected to review by a second evaluator.

## Results

The first section asked for descriptive information of the respondent, including personal characteristics, background of education, as well as discipline and expertise in the field by research and clinical work. We included 92 respondents in our study, with a broad age range from 22 to 88 years, and about two thirds (66%) being female. The majority of the respondents were resident in Europe (72%; with Sweden, Germany, and the Netherlands most frequently represented), had a training in psychology (73%), and indicated most frequently a MSc (28%) or PhD (44%) as the highest degree obtained. All participants had experience in the field of ADHD and related disorders via clinical work, research, or both. Because experience in one of the domains was sufficient to be included in our study, clinical experience (in years) and research experience (in number of publications), ranged broadly across participants from no experience at all (no clinical experience or no publication) to up to 40 years of clinical experience and more than 40 publications.

The second section of the survey focused on the current standard of impairment assessment in the work of the respondents (up to 71 participants responded to this section, see Table 3–5). Participants described *impairment* in this context (Table 3) mostly broadly as any interference with Activities of Daily Living (ADL; *N* = 37; 63%), and in several cases by emphasizing the academic and occupational context (*N* = 8; 14%). A few respondents (*N* = 4; 7%) referred to a discrepancy between the individual’s functioning and a reference standard (such as own or societal expectations), coping and stress (*N* = 3; 5%), cognitive deficits (*N* = 3; 5%), and quality of life issues (*N* = 2; 3%). The vast majority of respondents saw the purpose of impairment assessment in supporting diagnosis (*N* = 63; 89%), determining treatment needs (*N* = 56; 79%), and/or creating a treatment plan (*N* = 41; 58%). Also, more than a third (*N* = 28; 39%) of respondents indicated to use impairment assessment to evaluate treatment needs and establish congruence/divergence with symptoms. About 75% (*N* = 53) of the respondents reported to use more than one instrument for the impairment assessment in adults with ADHD, which are presented in Tables 4 and 5 with respect to their category, frequency of mentioning, rating, and associated strengths and weaknesses.

**Table 3.** Current Clinical Standard of Impairment Assessment and Need for Adaptation.

	*N*	% of total
What do you understand under the term “impairment” in adult ADHD? (*N* = 59)		
Issues with ADLs	37	62.7
Issues with ADLs, with a focus on academic/occupational difficulties	8	13.6
Discrepancy between daily life functioning and a reference standard (own expectations/societal expectations/ intellectual abilities/population norm)	4	6.8
Coping/distress in daily life	3	5.1
Cognitive deficits in daily life	3	5.1
Quality of life issues	2	3.3
Discrepancy of functioning	1	1.7
Comorbidity development	1	1.7
For which purpose do you assess impairment in the assessment of adult ADHD? (*N* = 71)		
Supporting diagnosis	63	88.7
Determining treatment needs	56	78.9
Creating treatment plan	41	57.7
Evaluating treatment response or resistance	28	39.4
Establishing congruence or divergence with symptoms	28	39.4
Other^a^	11	15.5
Which instruments/approaches are you applying for the assessment of impairment in adults with ADHD? (*N* = 70, see Tables 4 and 5)		
One instrument	17	24.3
Two instruments	9	12.9
Three instruments	21	30
More than three instruments	23	32.8
Do you think there is a need for adaptations or even new developments for a better impairment assessment in adult ADHD? (*N* = 54)		
No, no need to change current practice	3	5.6
A bit, minor adaptations needed	5	9.3
Much, considerable adaptations and/or new development needed	28	51.8
Very much, substantial adaptations and new development needed	18	33.3

a Other = determining legal disability status, research purposes, determining work/function ability, differential diagnosis, social legal assessment, determining need for academic accommodations.

**Table 4.** List of Reported Instruments for Impairment Assessment in Adult ADHD and Their Ratings (Total *N* = 70).

Instrument	*N*	%	*N* “poor/moderate/decent”	*N* “good/excellent”
Diagnostic criteria and structured interviews				
Interview (not specified)	25	35.7	8	9
Diagnostic interview for ADHD in adults (DIVA)	19	27.1	7	12
Homburger ADHD Scales for Adults (HASE)	3	4.3	0	2
Diagnostic classification systems (DSM/ICD)	3	4.3	2	0
Integrated Diagnosis of Adult ADHD (IDA-R)	1	1.4	1	
Mini international neuropsychiatric interview (MINI)	1	1.4	0	0
Conners’ Adult ADHD diagnostic interview for DSM-IV (CAADID)	1	1.4	1	0
Instruments to assess comorbid conditions, such as tic, autism, etc.	1	1.4	0	0
Functional impairment scales				
Weiss Functional Impairment Rating Scale (WFIRS)	9	12.9	5	4
World Health Organization disability assessment schedule (WHODAS)	4	5.7	1	3
Adaptive behavior assessment system (ABAS)	4	5.7	0	3
Impairment Rating Scale (IRS)	2	2.9	2	0
Academic Impairment Measure (AIM)	2	2.9	0	2
Adaptive Behavior Scale (ABS)	1	1.4	0	1
Rating Scale of Impairment (RSI)	1	1.4	0	1
Sheehan’s Disability Scale (SDS)	1	1.4	0	1
ADHD symptom self-report scales				
Conner’s Adult ADHD Rating Scale (CAARS)	9	12.9	3	4
ADHD Rating Scale (ASRS)	6	8.6	2	2
Wender Utah Rating Scale (WURS)	2	2.9	2	
Homburger ADHD Scales for Adults (HASE)	2	2.9	0	2
Integrated Diagnosis of Adult ADHD (IDA-R)	1	1.4	1	
Other self-rating scales of functioning				
Questionnaires (not specified)	8	11.4	2	3
Behavior Rating Inventory of Executive Function—Adults (BRIEF-A)	6	8.6	2	2
Barkley Scales	6	8.6	3	3
Patient Health Questionnaire—4 (PHQ4)	1	1.4	1	0
Achenbach System of Empirically Based Assessment for Adults (ASEBA)	1	1.4	0	1
Strengths and Difficulties Questionnaire (SDQ)	1	1.4	0	1
Self-assessment rating scale (not specified)	1	1.4	0	0
Behavioral observations and ratings (clinician, parents, and teachers)				
Observations (not specified)	3	4.3	2	1
Global Assessment of Functioning (GAF)	1	1.4	0	1
Child Behavior Checklist (CBC)	1	1.4	0	1
Clinical Global Impressions (CGI)	1	1.4	0	1
Performance assessment of cognition and intelligence				
Psychological/cognitive tests (not specified)	8	11.4	2	4
Intelligence tests	5	7.1	1	3
Testbattery of Attention Performance (TAP) or Vienna Test System (VTS—WAF)—Vigilance	4	5.7	1	0
Test of Variables of Attention (TOVA)	2	2.9	1	1
Conners’ Continuous Performance Test 3 (CCPT-3)	2	2.9	2	0
Objective records of impairment				
Report cards, academic performance, medical records, etc.	7	10	3	4
Quality of life and well-being				
Adult ADHD Quality of Life (AAQL)	1	1.4	0	1
EuroQol-5 Dimension (EQ5D)	1	1.4	1	0
Validity testing	1	1.4	0	1

*Note*. Not all participants rated their instruments so *N* regarding instruments and *N* regarding rating may not match.

**Table 5.** Strengths and Weaknesses of the Various Instruments for Impairment Assessment in Adult ADHD.

	Strengths	Weaknesses
Diagnostic criteria and structured interviews	• Flexible and comprehensive • Efficient and structured • Easy to administer and involve informants • Validated and standardized • Widely used	• Subjective/prone to bias • Difficult to interpret • Hard to obtain and time consuming • Requires extensive training • Culture/age considerations • Potential for faking
Functional impairment Scales	• Relevant • Comprehensive • Normative data • Easy, quick, applicable, and inexpensive • Culture appropriate • Reliable	• Subjective and hard to interpret • Potential for faking • Limited normative data • Not specific to ADHD • Time consuming • Age limitations
ADHD symptom self-report scales	• Comprehensive and in line with symptoms • Normative data • Widely used • Effective • Differential diagnosis	• Lack of cultural sensitivity • Vague/subjective • Low specificity • Potential for faking
Other self-ratings of functioning (see Table 4)	• Normative data • Comprehensive • Time effective • Good sensitivity/specificity • Widely used • Informant versions available	• Potential for exaggerating • Poor quality/not validated in some countries • Dependent on cognitive skill • Ambiguous/subjective • Reliance on parental reports • Not specific to ADHD
Behavioral observations and ratings (clinician, teachers, and parents)	• Widely used • Comprehensive • Normative data • Diagnostic plausibility	• Subjective reports • Limited focus on domains • Cultural variations • Limited to external behavior
Performance assessment of cognition and intelligence	• Sensitivity and validity • Cultural standardization • Comprehensive • Differential diagnosis • Provides performance measures	• Lengthy • Limited to current deficits • Poor specificity • Difficulty in pattern recognition • Time consuming • Not specific to ADHD
Objective records of impairment	• Objective • Different points of view • Collateral information • Comprehensive	• Not always available • Specificity of report cards • Inability to clarify information • Dependence on evaluators • Dependent on skill of observant
Quality of life and well-being	• ADHD-related domains • Easy to use • Quick administration	• Long • Difficult scoring • Lack of precision
Validity testing	• Objective evaluation • Well-developed • Large empirical database	• High subjectivity in test selection • Some tests poorly developed or with a weak empirical base • May be difficult to distinguish between true/false positives

*Note*. Selection based on frequency of answers.

Finally, the third section addressed the potential need for new developments with respect to better functional impairment assessment in adult ADHD and related disorders (see Tables 6 and 7, with up to 51 respondents). About 85% (*N* = 46) of the respondents indicated that *much* or *very much* adaptations or even new developments for a better impairment assessment in adult ADHD are needed (see last item on Table 3). The most important domains of functioning in the impairment assessment, as mentioned in open answers and presented in Table 6, are daily life functioning in general (*N* = 41; 91%), social and family relations (*N* = 38; 84%), and cognition (*N* = 34; 76%). Academic performance, occupational functioning, and reckless/dangerous behavior were also important as brought up by 56% (*N* = 25; academic and occupational functioning) or 42% (*N* = 19; reckless/dangerous behavior) of the respondents. Inspecting important aspects for a validation and perceived suitability of assessment approaches (Table 7), we see that good population norming, specificity for adult ADHD, and association to the burden of disease, stand out with each more than 80% (*N* ≥ 41) endorsement. Regarding assessment approaches, objective indication of impairment, and the consideration and integration of multiple sources and factors, were considered most suitable for a potential new instrument of a better impairment assessment, with more than 90% (*N* = 47) endorsement (Table 7).

**Table 6.** Domains of Importance for Valid Impairment Assessment (*N* = 45).

	*N*	*N* (%)
Daily functioning	41	91.1
Social/family relations	38	84.4
Cognition	34	75.6
Functioning in occupation	25	55.6
Academic performance	25	55.6
Reckless/dangerous behavior	19	42.2
Comorbidities	14	31.1
Mood	12	26.7
Health	8	17.8
Financial	8	17.8
Time management	7	15.6
Self-image	4	8.9
Sleeping patterns	2	4.4
Other	14	31.1

*Note.* Participants could select more than one domain, thus the total number of responses for all participants who filled in the question is *N* = 251. “Other” includes a great variety of open answers, including evaluation of qualitative studies, longitudinal assessment, assessment methods that do not require clinician training, and incorporating multiple disciplines.

**Table 7.** Important Considerations for Instruments Assessing Impairment in Adult ADHD (*N* = 51).

	*N* “much/very much”	*N* (%)
How important do you consider the following aspects for validation?		
Quick administration	25	49
Automatic scoring	28	54.9
Good population norms	45	88.2
Specific for disorder (adult ADHD)	41	80.4
Sensitive to change	34	66.6
Association or dissociation between impairment domains	33	64.8
Association or dissociation to symptom severity	38	74.5
Association or dissociation to burden of disease	41	80.4
How suitable do you find the following assessment approaches?		
Self-report	33	64.7
Other-report	42	82.4
Discrepancy between sources of information	38	74.6
Different scaling: counting frequency instead of subjective severity rating	30	58.8
Objective indications (e.g., factors or events) instead of subjective evaluation	47	92.2
In-the-moment assessment	27	62
Consideration and integration of multiple sources and factors	47	92.1

*Note.* Order of presentation in table corresponds to the order of presentation in the survey.

In additional exploratory analyses, we wanted to gain insight into the question whether the evaluation on the current form of impairment assessment and potential need for new developments differed between those who primarily work in research and those who follow clinical work. For this reason, we applied an operational definition of those who spent at least one third of their working time in one of the fields, with max 10% in the other and vice versa, with *N* = 10 researchers (*M* = 72.2% working time spent in research) and *N* = 43 clinicians (*M* = 90.7% working time spent in clinical practice). Tables 8 and 9 contrast key responses of both groups. Results have to be interpreted with caution because of small group size, imbalanced characteristics, and post hoc nature. Based on explorative data inspection, no marked differences in the evaluation of clinicians and researchers are observed.

**Table 8.** Explorative Comparison of Primary Clinicians and Primary Researchers: Instruments and Need for Adaptation.

	Clinicians (%)	Researchers (%)
Which instruments/approaches are you applying for the assessment of impairment in adults with ADHD? (*N* = 33 clinicians, *N* = 5 researchers)		
One instrument	7 (21.2)	1 (20)
Two instruments	5 (15.1)	2 (40)
Three instruments	9 (27.3)	2 (40)
More than three instruments	12 (36.4)	0 (0)
Do you think there is a need for adaptations or even new developments for a better impairment assessment in adult ADHD? (*N* = 24 clinicians, *N* = 5 researchers)		
No, no need to change current practice	1 (4.1)	0 (0)
A bit, minor adaptations needed	2 (8.3)	1 (20)
Much, considerable adaptations and/or new development needed	13 (54.3)	3 (60)
Very much, substantial adaptations and new development needed	8 (33.3)	1 (20)

*Note.* Numbers of respondents per question varies as some participants have skipped certain questions.

**Table 9.** Explorative Comparison of Primary Clinicians and Primary Researchers: Considerations for Instruments Assessing Impairment in Adult ADHD.

	Clinicians	Researchers
	*N* “much/very much” (%)	
How important do you consider the following aspects for validation?		
Quick administration	7 (29)	4 (100)
Automatic scoring	13 (59)	3 (75)
Good population norms	19 (86)	3 (75)
Specific for disorder (adult ADHD)	20 (91)	3 (75)
Sensitive to change	15 (68)	3 (75)
Association or dissociation between impairment domains	13 (59)	2 (50)
Association or dissociation to symptom severity	16 (73)	2 (50)
Association or dissociation to burden of disease	18 (82)	3 (75)
How suitable do you find the following assessment approaches?		
Self-report	13 (59)	4 (100)
Other-report	18 (82)	4 (100)
Discrepancy between sources of information	15 (68)	3 (75)
Different scaling: counting frequency instead of subjective severity rating	11 (50)	2 (50)
Objective indications (e.g., factors or events) instead of subjective evaluation	20 (91)	4 (100)
In-the-moment assessment	8 (36)	1 (25)
Consideration and integration of multiple sources and factors	19 (86)	4 (100)

*Note. N* = 22 clinicians, *N* = 4 researchers. Order of presentation in table corresponds to the order of presentation in the survey.

## Discussion

The present survey consulted an international group of from research and clinical practice on their understanding of functional impairment in the assessment of adult ADHD, their current practice, and potential need for adaptations or new developments.

The respondents of the present survey showed a clear and rather uniform understanding of the term and described *impairment* as any interference with activities of daily living, including the academic, occupational, and social setting. Further, impairment was defined as the inability to meet a reference standard, such as individual, social, or normative expectations, or one’s own ability levels, in various aspects of daily living. A number of respondents explicitly mentioned issues with stress, coping, and cognitive deficits in daily life. Our participants emphasized the importance of a thorough assessment of functional impairment, as evidenced by their utilization of multiple instruments for various purposes, including the diagnostic process, determining treatment needs or creating a treatment plan, or evaluating treatment response, also in association with symptoms.

Seventy participants provided responses regarding the utilization of instruments, their evaluation, and the associated strengths and weaknesses. Most frequently mentioned instruments refer to diagnostic criteria as assessed with structured interviews or ADHD symptom rating scales. Next to interviews that are not specified (36%), the DIVA (27%), CAARS (13%), and ASRS (9%), were most frequently named. While structured interviews and standardized and normed symptom rating scales have certainly their merits in guiding the diagnostic process and arrive at clinically relevant information on the presence and severity of ADHD symptomatology, they have significant limitations when employing for a comprehensive assessment of functional impairment. Some of the instruments make an exception as they prompt the examiner in evaluating functional impairments in several aspects of daily living (such as the DIVA). These limitations correspond to the concerns in the literature stressing that the reliance on the assessment of ADHD symptoms (in structured interviews and/or rating scales) bears the risk of overlooking the impairment criterion of ADHD (Nelson et al., 2014; Weis et al., 2019), and, hence, may result in inflated numbers of false-positive ADHD diagnoses (Gathje et al., 2008; Gordon et al., 2006). Further, the reliance on ADHD symptoms, and neglecting aspects of functional impairment, may overestimate treatment response as a sizeable number of individuals remain functionally impaired after symptom remission (Sasser et al., 2016; Weiss, 2022). Specific instruments for the functional impairment assessment are mentioned by about a third of the respondents completing this question, with the measures WFIRS (as a tool for relative impairment; 13%) and WHODAS (measuring absolute impairment; 6%) standing out to be mentioned most frequently. The prominent role of the WFIRS in the functional impairment assessment as indicated by our respondents is in line with current guidelines and opinion papers in which the WFIRS is put in the foreground (e.g., Sibley, 2021; Weiss, 2022). Additionally, the respondents of this survey indicated self-ratings on other domains of functioning (e.g., executive dysfunction as assessed with the BRIEF-A), referred to Barkley’s scales (exist with regard to various aspects of functioning, including functional impairment, executive dysfunction, and ADHD symptoms), and included behavioral observations by others. Interestingly, ICF core sets were not mentioned by our respondents in the present survey. Despite significant milestones on the ICF regarding a conceptual framework of impairment, and defining relevant domains of functioning (Bölte et al., 2018, 2024; De Schipper, Mahdi, et al., 2015; de Schipper, Lundequist, et al., 2015; Mahdi et al., 2018), one may conclude from the results of our survey that clinicians and researchers may still have limited access to the ICF framework or do not know how to include it in their assessments.

Moreover, several respondents of this survey also considered neuropsychological tests for cognition (in particular attention) and intelligence as suitable means to determine functional impairment in adults with ADHD. The use of cognitive performance tests for the assessment of functional impairment is particular remarkable as they have been criticized prominently for lacking external validity and inadequately reflecting day-to-day behavioral issues (for a discussion, see Barkley, 2019; Barkley & Fischer, 2011; Fuermaier et al., 2015). Objective records of impairment (e.g., academic failure and medical records) were mentioned by seven respondents, with two references to quality of life and one to validity testing.

Of note, the respondents signaled their awareness of the constraints in using these instruments for the assessment of functional impairment. The dissatisfaction of the respondents with the current status of impairment assessment is reflected in the poor, moderate, or at max decent ratings of the various instruments. While respondents acknowledge the comprehensive choice, widespread use, straightforward administration, status of validation with regard to psychometric properties, and availability of normative data, the instruments have been repeatedly criticized for their difficult, vague, or subjective interpretation, not being specific to ADHD, insufficient consideration of age and cultural background, and considerable time consumption in administration and scoring.

Building upon the constraints of the available instruments discussed above, 46 of 54 respondents believe that considerable or substantial adaptations or new developments are needed for a better assessment of functional impairment in adult ADHD, and, in contrast, only three respondents did not see any reason for change in current practice. The most important domains for adequate assessment of impairment are daily functioning (91%), social and family relations (84%), cognition (76%), and occupational functioning and academic performance (both 56%). For validation of new instruments, respondents stated most frequently the need for good population norms, specificity for adult ADHD, and the association/dissociation to the burden of the disorder. Finally, more than 90% of the respondents value objective assessments (over subjective evaluations) and the integration of multiple sources of information in the assessment of functional impairment in adult ADHD.

### Limitations

The results of this survey must be seen in the context of limitations. One of the major limitations is certainly the selection bias of our sample of participants. It must be considered that our survey addresses predominantly clinicians and researchers who are known for their expertise (and are therefore invited to take part), attend international conferences such as the World Congress on ADHD (primary event in which potential participants were approached), feel comfortable in English language (which was the administration language of the survey), and eventually have time to take part in research of this kind. This selection of participants may have biased the responses and, hence, the conclusions of this study. Related to this point, the results are certainly influenced by an imbalanced sample composition, with the majority being resident in Europe, and the vast majority having a background in psychology (hence, the survey included a disproportionally small number of psychiatrists). Related to the selection bias, it must be noted that our sample included participants from research and/or clinical practice at different stages of their careers. The purpose and requirement of impairment assessment in research may differ from those of clinical practice. We employed an operational definition of researchers and clinicians, and observed no marked differences between the evaluations of both groups in explorative data inspection. This issue needs to be followed in systematic follow-up research to draw firm conclusions.

## Conclusions

The diagnostic criteria for adult ADHD require the presence of impairment in more than one domain of functioning. This criterion is indispensable as the experience of functional impairment serves as a fundamental requirement for mental disorders (Spitzer et al., 2018). This stipulation lowers the risk for overdiagnoses, and facilitates accurate treatment outcome evaluation. However, the present study demonstrates that clinicians and researchers do not feel well equipped for this assessment, as they have access to only a few instruments that address functional impairment in adult ADHD adequately. As a consequence, clinicians and researchers use a wide range of alternatives for an assessment of functional impairment, of which they know that they are imperfect and are associated with various shortcomings. We advocate for research endeavors aimed at either refining existing tools, or devising new measures, that help clinicians and researchers to determine the presence and extent of functional impairment in adult ADHD and related disorders. Efforts should also be invested in the dissemination of these instruments to support widespread access, as well as easy and efficient administration, scoring, and interpretation. An assessment that is based less on subjective evaluation but more on objective indications of impairment, and that integrates multiple sources of information, is advocated by the great majority of the respondents.
